# Variations in dietary patterns in the ancient Greek colony of Abdera: insights from isotopic evidence and Bayesian modelling

**DOI:** 10.1007/s12520-025-02242-2

**Published:** 2025-06-14

**Authors:** Zisis Anastasios, Georgiadou Angeliki, Ganiatsou Elissavet, Xanthopoulou Panagiota, Kallintzi Constantina, Papageorgopoulou Christina

**Affiliations:** 1 Laboratory of Biological Anthropology, Department of History and Ethnology, Democritus University of Thrace, Komotini, Greece; 2 Ephorate of Antiquities of Xanthi, Greek Ministry of Culture, Emeritus Ephor of Antiquities, Archaeological Museum at Abdera, Abdera, Greece

**Keywords:** Bayesian modelling, Ancient Greek colonization, Infant feeding practices, Stable isotopes of Sulphur, Stable isotopes of Carbon and Nitrogen, Ancient migration and mobility, WARN

## Abstract

Abdera is an ancient Greek colony in northern Aegean. It exhibits a unique foundation history as it was first established in 654 BC by the Ionian city of Klazomenae and in 545 BC by the city of Teos. The first colonial endeavor failed due to harsh living conditions and conflicts with local populations. Exposed to unfamiliar challenges, the settlers faced physical strain and maladies, particularly affecting the subadults, who were deprived of proper care and nutrition during critical periods of life. After about a century, the city of Teos colonized Abdera under the pressure of the Persian attacks. The new colonial endeavor was successful, and the city managed to capitalize on its natural resources, flourishing through the centuries. This study reconstructs the diet of 109 adults and subadults from Abdera dating from the Archaic through the Roman times (654 BC–400AD) using stable isotope ratios of carbon (δ^13^C), nitrogen (δ^15^N) and sulphur (δ^34^S) from bone collagen. Bayesian modelling was implemented to quantify the relative consumption of different food sources in Abdera and compare it with other contemporary sites. Weaning duration was estimated to investigate the nutritional and health status of infants that is believed to have affected the fitness of the population in the long run. Our results indicate that the first settlers of Abdera primarily relied mostly on C_3_ plants, freshwater fish and less animal protein, while marine fish and C_4_ plants (millet) complemented the diet. This pattern persisted over time. Bayesian modelling revealed differences in consumption patterns within the site and among other contemporary populations. The first colonization phase was characterized by prolonged weaning indicating that harsh living conditions forced mothers to rely more on breastmilk as a nutritional buffer. The sulfur analysis revealed different migration and dietary patterns in Abdera females especially during the first colonial phase. Our study highlights the significance of diet as a key factor for studying the trajectory of a settlement, and for understanding the growth, the resilience, and the cultural evolution of the ancient Greek colonies.

**Supplementary Information:**

The online version contains supplementary material available at 10.1007/s12520-025-02242-2.

## Introduction

The Greek colonial expansion of the early 1st millennium BC spread people, goods, art, ideas, and lifestyles across the Mediterranean and the Black Sea. Greeks from the Aegean Islands, Asia Minor and mainland Greece embarked on numerous expeditions (Graham 1964; Ridgway 1992; Boardman 1999; Grammenos and Petropoulos 2003; Tsetskhladze 2006). Their search for new homes was instigated by many reasons: internal strife, social conflicts, political strategies, famine or poverty (Graham 1964; Ridgway 1992; Boardman 1999; Grammenos and Petropoulos 2003; Tsetskhladze 2006), demographic pressure (Sallares and Gomzi 2001; Scheidel 2003), new land to farm, more livestock to own, ampler natural resources to exploit and new markets to expand at (Soueref 2011; Purcell 2005; Gimatzidis 2022). *Emporia* (trading posts) were in some cases the predecessors of colonies, whereas from the mid-8th c. BC the Greek *poleis* (city-states) started to expand with more targeted, longer-term intentions. The founding of hundreds of new cities in a remarkable variety of areas, the interaction of the settlers with heterogeneous indigenous populations and the relatively short time in which the colonies evolved into major cultural, commercial, and political centres has turned Greek Colonisation into one of the most influential themes in European ancient history and archaeology.

Varying aspects of the impact of the Greek Colonisation have been studied by scholars: Mediterranean networks that persist for centuries (Braudel 1972), the emergence of Greek identity (Malkin 2003; Hall 2009), the model of core-culture and periphery demonstrating the impact of Greek civilisation on natives (Rowlands et al. 1987), and the opposite view demonstrating the impact of the natives on the Greek civilisation (Stoddart 1989; Whitehouse and Wilkins 1989). Plenty of historical and archaeological studies have tried to outline the patterns that were followed during these expeditions (Graham 1964; Ridgway 1992; Boardman 1999; Grammenos and Petropoulos 2003; Tsetskhladze 2006).

Albeit the long scholarly investigations on Ancient Greek Colonization, relatively little is known about the bioarchaeology of this process, including the case of Abdera. This is a paradox, considering that the study of living conditions in colonial settings can reveal significant aspects of the migratory process. At least during the initial phases, life in the colony should have been challenging, due to the need of a population to adapt to new conditions, which usually lacked the infrastructure of an organised polis. However, the number of bioarchaeological studies on the topic is still limited (see Zisis and Papageorgopoulou 2019) for a review). Exceptions are the necropolis of Metaponto in Italy (Henneberg and Henneberg 2003; Henneberg and Henneberg 2001), Apollonia Pontica in Bulgaria (Keenleyside 2008, 2011; Keenleyside et al. 2006) and Apollonia pros Epidamnon, in modern-day Albania (Kyle et al., (2016). The study of Kyle et al., (2016), for example, describes an increase of physiological stress of the post-colonial population, due to impoverishment after Corinth’s extraction of local resources, changes of sanitation and disease transmission associated with the new living conditions of large-scale urbanism.

In our study, we approach Greek colonization through stable isotope analysis, an established and valuable method for studying paleodietary and socioeconomic history (Heinrich et al. 2021). We reconstruct diet from the Archaic period to the Roman times (c. 7th c. BC to 4th c. AD) in the ancient city of Abdera, a renowned Greek colony in Aegean, aiming to describe the subsistence of the colony. Our approach offers valuable insights into the population’s overall fitness through time, the socio-cultural differentiations between sexes and age-groups, and the role of infant diet in the development of the site from colony to a city.

Towards this end, we focus on the analysis of carbon, nitrogen and sulphur stable isotopes in bone collagen. Carbon isotopic analysis enables the differentiation between C_3_ and C_4_ plants (DeNiro and Epstein 1978). Nitrogen isotope ratios (δ^15^N) are mainly used to distinguish between herbivorous and carnivorous consumers in terrestrial ecosystems, while both δ^13^C and δ^15^Ν are used to characterize marine vs terrestrial protein (Ambrose 1990). Sulphur isotopes in bone collagen are valuable indicators for detecting freshwater or marine protein intake. Furthermore, they are linked to local geology and can be useful for the study of residence and migration (Nehlich 2015). We analyzed the stable isotopes in adult long bone collagen as their values reflect food consumption over approximately 10 years before death (Fahy et al. 2017) and in subadult long bone collagen to reconstruct the breastfeeding and weaning duration (Herring et al. 1998; Tsutaya and Yoneda 2015) in order to delve deeper in the diet of the subadult population. Infant nitrogen ratios during exclusive breastfeeding show increased values compared to their mothers (Fogel et al. 1989). Subadults with higher δ^15^N values than the adult population are considered as individuals that are still weaning, while those with similar values as fully weaned (Halcrow et al. 2021).

We also utilized the approach of Bayesian isotope mixing models to produce probabilistic estimates of dietary proportions. Bayesian inference has penetrated the theory of isotope mixing models, which consider that the isotopic values of individuals derive from the admixture of different dietary sources, such as animal protein, plants, fish etc. (Cheung and Szpak 2021). By supplying the isotopic values of the dietary sources, it is possible to estimate their relative contribution in the diet (Stock et al. 2018). Bayesian models are also used for weaning reconstructions using isotope measurements of bone collagen (Tsutaya and Yoneda 2013). WARN calculates the age at the beginning and the completion of weaning and overcomes common problems of subadult bone collagen analysis. Specifically, the different turnover rates of bone collagen in infants and adults, the individual variations in ^15^N enrichment from maternal to infant tissues and the diversity of the δ^15^N composition of weaning foods (Tsutaya and Yoneda 2013).

Using this modelling approach, we estimate the relative proportions of the diet in Abdera, as well as in six other ancient cities (Apollonia Pontica, Athens, Thebes, Helike, Edessa, and Knossos), spanning from the Archaic to Roman periods. Our aim is to compare the utilization of resources, the accessibility, and the provisioning of food within the distinct socio-cultural and environmental contexts of each chronological period. This research pioneers the study of human subsistence during the second Greek colonization. We contribute systematic results of a large anthropological sample from Aegean Thrace at the dawn of the Greek city-state formation and the expansion of the ancient Greek civilization using cutting edge analytical tools and targeted archaeological data.

## Materials and methods

### The city of Abdera, its historical importance and bioarchaeological context

**Fig. 1:**
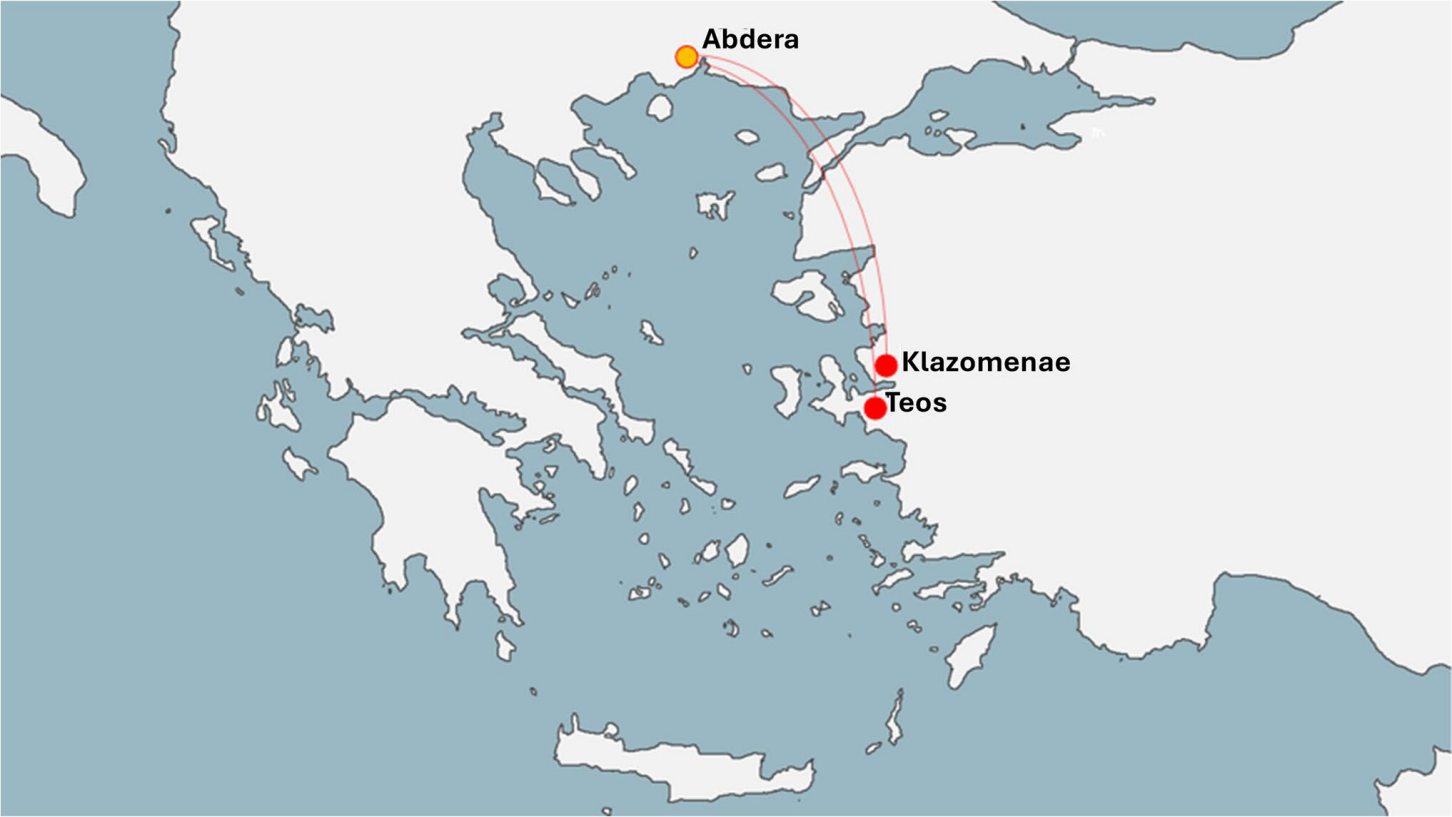
Map of Greece with the colony-Abdera (yellow dot) and the mother-cities Klazomenae and Teos (red dots)

The ancient city of Abdera (40.9815562, 24.9515371) is situated approximately 6 km south of its modern-day counterpart on the Thracian coast (Fig. 1). Abdera was founded in a geographically privileged position, with the sea in the south, Nestos River in the west, and Lake Vistonis in the east. The economy of Abdera was based on trade, agriculture (especially on the cultivation of grapes and cereals), fishery and livestock (Kallintzi et al. 2022). Moreover, Abdera’s thriving economy during the Classical period (490/80–323 BC) was developed due to the exploitation of natural resources (Loukopoulou 2004). The historian Diodorus (1st c. BC), referring to events from 408 BC, describes Abdera as one of the most powerful cities in Thrace (Diod. XIII 72, 1–2).

The unique historical occasion regarding Abdera is the city’s twofold foundation by two different though neighboring cities of Asia Minor. The first attempt was made *ex nihilo* (*i.e.,* no pre-existing settlement is known at the location) by Ionians from Klazomenae (c. 654 BC). As the Clazomenian endeavor was not successful, the city was re-founded about one century later (545 BC) by the citizens of Teos, who fled to Abdera in order to escape the Persian attacks (Graham 1992; Kallintzi 2011). What is particularly intriguing about this colonisation is the entire town relocation to Abdera, with a portion of the population later returning to Teos. This allowed Pindar to remark that Abdera became both the colony and the mother city of Teos. The Teians managed to maintain the colony against the pressure from the local Thracian populations, although the conflicts continued for centuries. Only much later, in the time that followed the Macedonian conquest (around 350 BC), cultural, economic, social and political homogenization was progressively established in the area (Kallintzi et al. 2021).

The archaeological research at Abdera began in 1950 by the Archaeological Society at Athens, while until 1973, the administrative responsibility of the area was held by the ΙΗ Ephorate of Prehistoric and Classical Antiquities (Kavala), and after 1973 by the ΙΘ Ephorate of Prehistoric and Classical Antiquities (Komotini). Since 2014, the archaeological site of Abdera is under the jurisdiction of the Ephorate of Antiquities of Xanthi. The Archaeological Society mainly excavated parts of the urban layout, whereas the Ephorates of Antiquities primarily conducted rescue excavations in the cemeteries, resulting in a wealth of material: tumuli, graves, grave markers, offerings, and osteological remains.

The city’s necropoleis have yielded large numbers of human remains from a diverse chronological range, beginning from its first foundation years and spanning to the late Byzantine times (Kallintzi 2006, 2011, Kallintzi 2004). The cemeteries of the Clazomenians were established and primarily used from the mid-7th to the first three decades of the sixth c. BC (Skarlatidou 2010). They have been identified at five locations: around the northern enclosure of the city, the first area settled by the colonists (Kallintzi 2004, 2011). The cemetery at Ammolofos has been excavated more extensively. The dead were either cremated or buried. Cremations were carried out on the surface of the ground or in pits. Burials were made in pit-shaped tombs or in cists (primarily using jars and amphorae).

During the late 6th to early fifth c. BC, the remaining Clazomenians living in the city of Abdera continued to use these cemeteries as identified by the archaeological research. Their tombs are mostly clay sarcophagi, as well as pit and cists graves, primarily with amphorae and are found in two areas (Kallintzi 2004). From the mid-6th to the third c. BC, the Teians, i.e. the second phase colonists, established a tumulus cemetery that covers an area of about 3,000 acres. The earliest tumulus graves date to the late 6th c. BC and the latest to the early 3rd century BC. Regarding burial practices, both cremation in rectangular pits and inhumations were employed. The latter occurs in almost all known types of tombs: stone and clay sarcophagi, chest-shaped, roofed, pit graves, and cists, jars and amphorae (Kallintzi 2006). Hellenistic cemeteries from the late 3rd century BC to the second half of the 2nd century BC are located to the west of the northern enclosure, which was then abandoned.

During the 3rd–4th c. AD, the western part of the southern enclosure near the gate and a large area to the west of it was destroyed by flood. These areas were abandoned and turned into a cemetery with mainly chest-shaped graves that often contain more than one person. This cemetery was initially pagan but was later converted to Christian and was used until the twelfth century. The individuals included in this study from the Roman period cemetery are dated to 200–400 AD.

### The skeletal material

A total of 109 individuals (62 adults and 47 subadults) was used in this study. The skeletal material analyzed originates from cemeteries of Abdera distributed as follows: 12 individuals identified as first-generation Clazomenian settlers (654–570 BC), 27 individuals classified as third- and fourth-generation Clazomenian inhabitants (late 6th–early 5th c. BC), and 22 individuals classified as first and subsequent generations of Teian settlers (late 6th c. BC–early 3rd c. BC), 29 individuals that date to the Hellenistic period (late 3rd c. BC–first half of 2nd c. BC), and 19 individuals date to the Roman period (200–400 AD). These groups are differentiated based on stratigraphy and archaeological context. Detailed information about the dataset can be found in Table S1 (Supplementary Material). Sex and age-at-death assessment was based on the observation of cranial and pelvic morphological features (Phenice 1969; Ferembach et al. 1980; Buikstra and Ubelaker 1994). The age-at-death estimation for the subadult population was based on teeth development according to Ubelaker (1978).

Four faunal samples from the site were analyzed to produce the reference isotope baseline of the site, necessary for the sulphur analysis. The animal samples originate from the species of equus (horse, donkey etc.). Horses and donkeys are not considered part of the population’s diet but were mostly used for traction. Therefore, their sulphur values reflect the local baseline as these species were most likely fed from local sources. Human sulphur values that can be considered as “local” are expected to range around this value, for a diet based primarily on local terrestrial protein.

### Sampling and collagen extraction

Collagen was extracted from femora and tibiae and, in the case of their unavailability, from humeri according to Brown et al., (1988), and modified by Richards and Hedges (1999). Approximately 300–500 mg of compact bone was surface cleaned using a diamond drill. The samples were demineralized at 0.5 M HCl at 4 °C for several days, rinsed to neutrality with double-distilled water, gelatinized at 72 °C for 48 h and then filtered using Ezee Filters (5–8 mm). The samples were frozen at −24 °C for at least 72 h and were freeze-dried for 48 h. Preparation of samples and collagen extraction was performed at the Laboratory of Biological Anthropology, DUTH (Greece) and delta values were measured at Curt-Engelhorn-Zentrum Archaeometrie gGmbH (CEZA) and Isoanalytical Ltd, UK. Samples were weighed out for duplicate runs. Systematic bias (u(bias)), precision (u(Rw)) and total analytical uncertainties (u_c_) were measured according to the equations reported in Szpak et al. (2017).

The quality of collagen was assessed using the percentage yield of collagen (> 1%,), nitrogen (N: 5–17%), carbon (C: 15–47%), and sulfur percentages (S: 0.15- 0.35%) as well as C/ N, C/ S, N/ S ratios (C/N: 2.9–3.6, C/S: 300–900, N/S: 100–300). Only samples that met the collagen quality criteria as reported in literature were included in this study (Ambrose 1990; DeNiro 1985; van Klinken 1999; Harbeck and Grupe 2009; Nehlich and Richards 2009)*.* We compared the distribution of δ^15^N, δ^13^C and δ^34^S between subadults and adults with Kolmogorov–Smirnov tests. Descriptive statistics and tests as well as data visualizations were performed in R (version 4.3.2).

### Weaning reconstruction using the Bayesian model WARN

To compare the nitrogen ratios of adult females and subadults, we plotted their values, and we also used the Bayesian model WARN in R (version 4.3.2). WARN estimates the beginning and the completion of weaning within the framework of Approximate Bayesian computation (ABC) (Tsutaya and Yoneda 2013), accounting for bone collagen turnover rate, which declines from infancy to puberty. The model uses the δ^15^N values of subadults and the mean ± SD δ^15^N values of females. WARN requires at least six subadult individuals for analysis, therefore it was performed only to chronological groups that included at least six subadults aged below 10 years of age.

### Diet reconstruction using the Bayesian model MixSIAR

The MixSIAR R package (Stock et al. 2018) was used to investigate probabilistic variations in dietary practices between chronological periods. The MixSIAR package employs Bayesian statistics to run mixing models using biotracer data (i.e. stable isotopes, fatty acids) and has the advantage of accounting for uncertainty associated with empirical or calculation errors (Galván et al. 2012; Moore and Semmens 2008). MixSIAR necessitates three data files to provide estimates: 1) the isotopic values of consumers 2) the potential dietary sources and 3) the trophic discrimination factors (TDFs) for each dietary source.

We used a 2-tracer (C, N) and 5-source approach (animal protein, C_3_ plants, C_4_ plants, freshwater fish, marine fish) (Table 1). Both trophic discrimination factors and isotopic values of dietary sources were taken from literature (Ambrose 2002; Dotsika et al. 2019; Vika and Theodoropoulou 2012). It is recommended that the number of dietary sources should be less or equal to the number of isotopic tracers + 1 (Phillips, 2001; Schwarcz, 1991; Stock et al. 2018), i.e., in the present study δ^13^C and δ^15^Ν. To ensure that the results are reliable we have run the model in the “short” version (chain length = 50,000, Burn-in = 25,000, thin = 25).

**Table 1:** Isotopic values and standard deviations (‰ AIR for nitrogen; VPDB for carbon) for probable dietary source components used in the MixSIAR computations for individuals from Abdera (654 BC –400 AD).; Source isotopic compositions and trophic discrimination factors (TDF) were taken from the literature (Ambrose 2002; Dotsika et al. 2019 Vika and Theodoropoulou 2012)

Sources	Source isotopic compositions (‰) and SDs				TDF (‰) and SDs			
	δ^13^C	SD	δ^15^N	SD	δ^13^C	SD	δ^15^N	SD
Animal protein	−19.0	1.0	6.2	3.0	3.6	1.2	4.5	0.5
C_3_ Plants	−25.0	1.0	2.0	1.0	3.6	1.2	3.3	0.9
C_4_ plants	−12	1.0	2.0	1.0	3.6	1.2	3.3	0.9
Freshwater fish	−20.8	4.8	12.4	3.4	3.6	1.2	4.5	0.5
Marine fish	−15.7	2.75	8.6	2.55	3.6	1.2	4.5	0.5

We examined the diet of the Abdera population across the different chronological periods to discern changes in subsistence as the site developed. To review diet in a broader spectrum, we used published δ^13^C and δ^15^N ratios from the Aegean and the Black Sea dating to the Archaic through Roman periods (Table 2). We used the isotopic values only of the adult population because the collagen values of subadult bone are influenced by breast milk consumption, long after the complete cessation of breastfeeding and this may lead to incorrect estimates especially for nitrogen (Fogel et al. 1989).

**Table 2:** Sites and number of included individuals (n = 265) used in this study for diet modelling with MixSIAR

Chronological period	Site	N	Reference
Archaic period	Abdera	14	*This study*
	Athens	2	Lagia 2015
	Apollonia Pontica	54	Keenleyside et al. 2006
Classical period	Abdera	6	*This study*
	Athens	14	Lagia 2015
	Thebes	19	Vika 2011
Hellenistic period	Abdera	11	*This study*
	Athens	20	Lagia 2015
	Thebes	28	Vika 2011
	Helike	5	Borstad et al. 2018
	Knossos	19	Moles et al. 2022
Roman period	Abdera	6	*This study*
	Athens	13	Lagia 2015
	Edessa	19	Dotsika et al. 2022
	Helike	8	Borstad et al. 2018
	Knossos	27	Moles et al. 2022

## Results

### Collagen quality control and analytical uncertainty

#### Stable nitrogen, carbon and sulphur ratios of humans from ancient Abdera

Out of the 109 samples, 71 yielded enough collagen and were measured for stable carbon and nitrogen, while 51 were measured for sulphur (Table 3). Individual isotopic ratios (δ^15^Ν, δ^13^C, δ^34^S), collagen yields (%), as well as elemental indicators (%C, %N, %S, C/N, C/S, N/S) are reported in Table S1. One sample (ABD 131) yielded low nitrogen and carbon content (2.9% and 8.6% respectively) and was excluded from further analysis. The remaining 70 samples are compiled with the published criteria for collagen integrity for carbon and nitrogen. Out of the 51 samples that were measured for sulphur, 46 met the quality assessment criteria (Table S1). Table S2 shows the descriptive statistics summarizing the dataset.

**Table 3:** The number of subadult (0–19 years old) and adult individuals (20–50 + years old) analysed in this study according to settlement phase and chronological period: first Clazomenian settlers: 654–570 BC, Next generations of Clazomenians: late 6th c.– early 5th c BC, First and next generations of Teians: late 6th c. BC–early 3rd c. BC, Hellenistic cemetery: late 3rd c. BC–first half of 2nd c. BC, Roman cemetery: 200–400 AD

	Subadults	Adults			Total
		Males	Females	Undet.	
First Clazomenian settlers	0	4	4	1	9
Next generations of Clazomenians	9	2	5	0	16
First and next generations of Teians	0	7	1	2	10
Hellenistic period Cemetery	5	7	4	2	18
Roman period Cemetery	11	3	2	1	17
**Total**	25	23	19	6	70

#### Stable nitrogen, carbon and sulphur ratios of fauna from ancient Abdera

Out of the four fauna samples that were analysed only two produced adequate material for analysis (Table S3). %C has an average of 41.4 ± 0.2 and %N has an average of 14.9 ± 0.1. The average value of δ^13^C is −19.6 ± 0.4‰, for δ^15^N values is 6.6 ± 0.1 and for δ^34^S is 8.3 ± 0.1‰. The two fauna samples represent the local archaeological baseline for sulphur. Therefore, we expect human values to range around this value for a diet based on local terrestrial protein.

#### Analytical Uncertainty

Following the recommendations of Szpak et al. (2017), precision (u(Rw)) was determined to be ± 0.1‰ for δ^13^C, ± 0.1‰ for δ^15^N, and ± 0.3‰ for δ^34^S, based on repeated measurements of calibration standards, check standards, and sample replicates. Accuracy or systematic error (u(bias)) was determined to be ± 0.1 for δ^13^C, ± 0.2 for δ^15^N, and ± 0.2 for δ^34^S, based on the difference between the observed and known δ values of the check standards and the long-term standard deviations of these check standards. The total analytical uncertainties are estimated to be ± 0.1 for δ^13^C, ± 0.2 for δ^15^N, and ± 0.4 for δ^34^S. The detailed calculations are provided in Tables S4, S5 and S6.

### Subadult population and weaning reconstruction

**Fig. 2:**
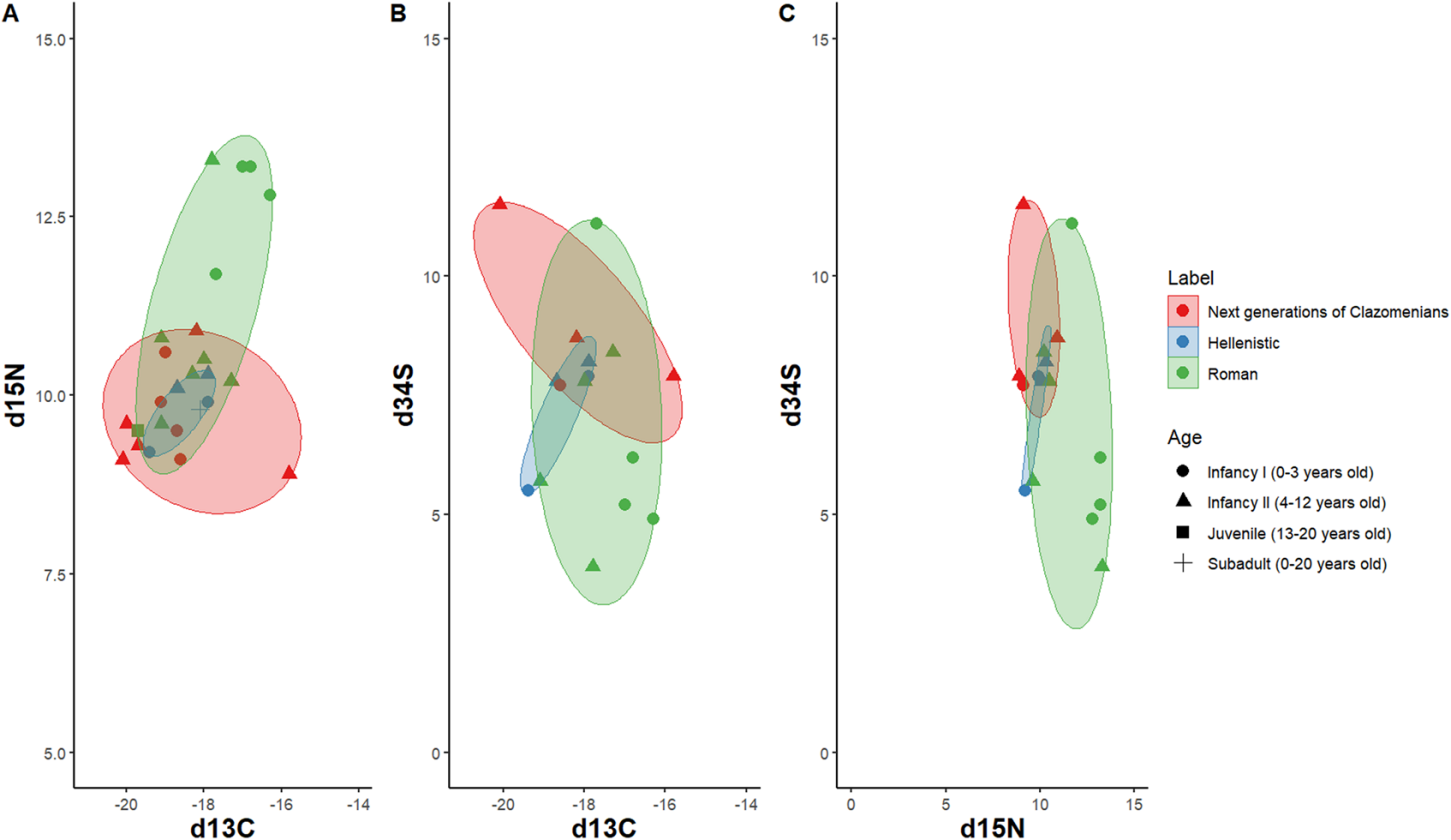
Scatterplots showing the spread of δ^13^C, δ^15^N and δ^34^S for Abdera subadult individuals (n = 25) according to age groups, settlement phase and chronological period: first Clazomenian settlers: 654–570BC, Next generations of Clazomenians: late 6th c.–early 5th c. BC, Hellenistic cemetery: late 3rd c. BC–first half of 2nd c. BC, Roman cemetery: 200–400 AD)

Figure 2A shows the δ^15^Ν and δ^13^C spread in subadults (n = 26) that range from 13.3‰ to 8.9‰ and from −15.8 to −20.1‰. Sulphur ratios were measured in 18 individuals spanning 16.4‰ to 3.9‰ (Fig. 2B and 2C). The subadult individuals from the first Clazomenian settlers and the group of Teians (both first and later generations) did not yield sufficient material for isotopic analysis. The next generation Clazomenians (n = 9) show limited variation in their nitrogen, carbon and sulphur ratios, with only few outliers (ABD94, ABD 93). The Hellenistic subadults (n = 5) form a uniform cluster within the first-generation Clazomenians. The Roman group exhibits the greatest variation in nitrogen, generally decreasing with age, except for ABD55 (aged 4–12 years), who has the highest nitrogen value (13.3‰). Additionally, this group includes more individuals with distinctly positive carbon ratios (greater than −17.5‰), which pattern is also evident in δ^34^S.

**Fig. 3:**
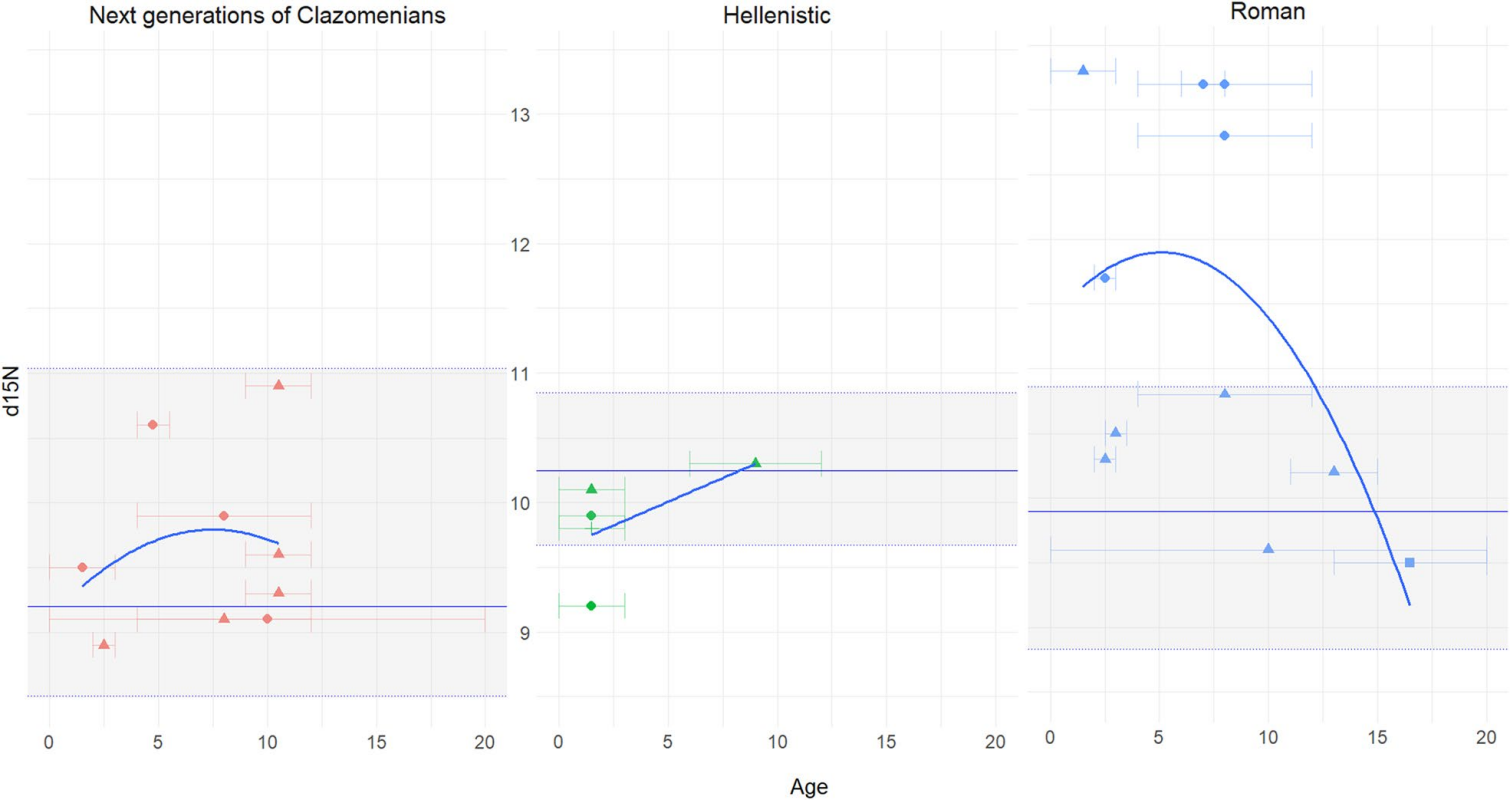
Stable nitrogen isotope values for Abdera subadults compared with mean nitrogen value for adult females (solid lines) and adult female range (dotted lines) with quadratic fit curve showing δ^15^N value trends related to with breastfeeding and weaning. X-axis indicating age in years

Figure 3 illustrates subadult δ^15^N, plotted alongside the mean δ^15^N value of adult females (first Clazomenian settlers: 4 ind., Next generations of Clazomenians: 5 ind., Hellenistic cemetery: 4 ind., Roman cemetery: 2 ind.). For subadults with a broad age range (e.g., 4–12 years), midpoint ages were used, with error intervals representing the age range. The δ^15^N values, characterized by a 2–3‰ enrichment relative to adult females is observed only among the Roman subadults. In all other cases, the subadult values are below or within the female adult range.

**Fig. 4:**
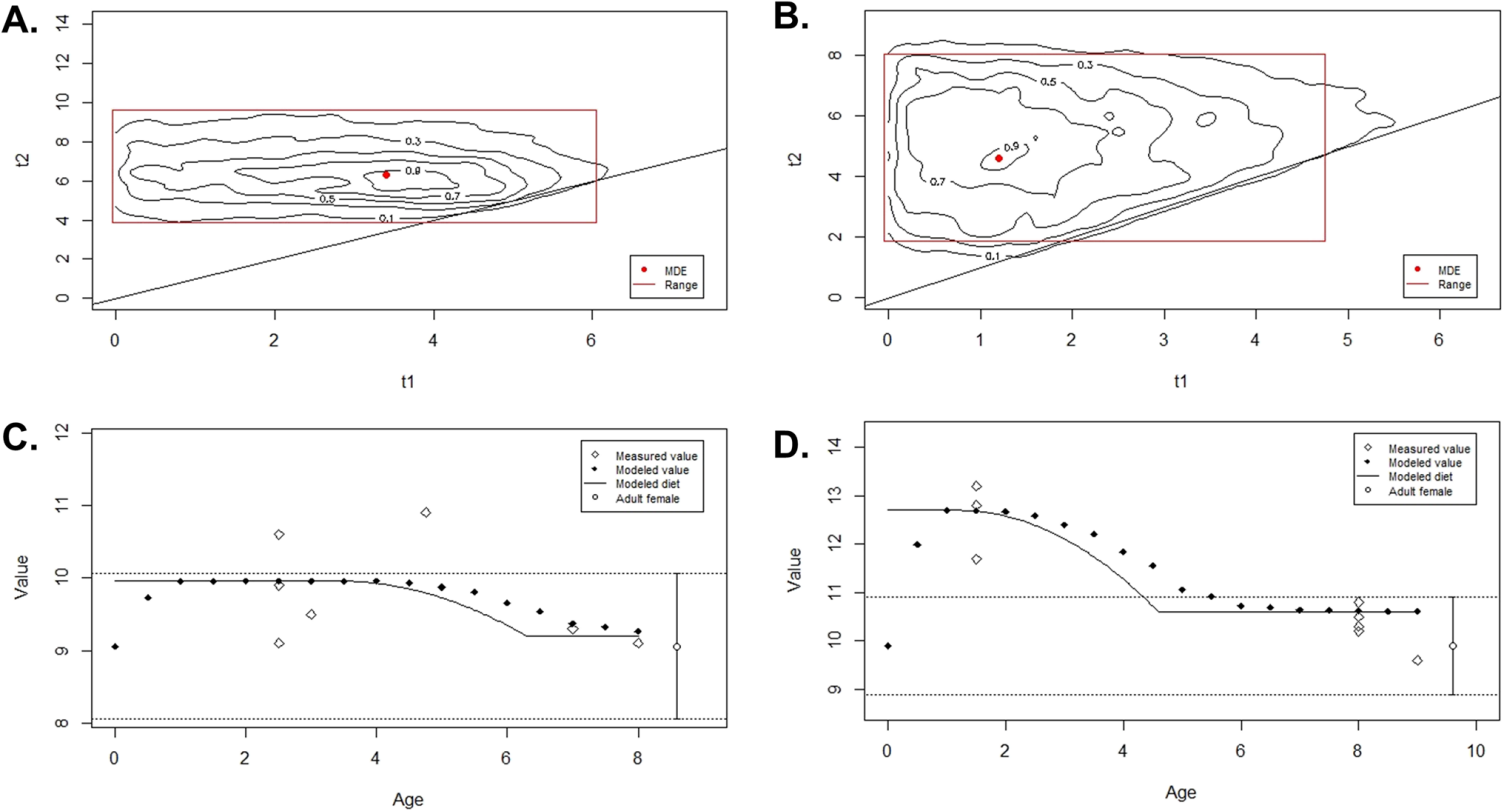
WARN model probability and 95% credibility interval of the beginning (t1) and ending (t2) ages of weaning presented in the red rectangle for the Abdera subadults and Transverse comparison of δ^15^N values calculated from reconstructed maximum density estimators (MDEs) by WARN using δ^15^N measurements. A and C include results for Abdera subadults dating to the Archaic period. B and D include results for Abdera subadults dating to the Roman period

Considering these results, we applied the WARN model to next generation of Clazomenian and Roman individuals (Fig. 4). The median age points used in the model are reported in Table S1. According to WARN, the maximum density estimations (MDEs) for the Clazomenian individuals indicate that weaning likely began at 3.4 years (marginal probability = 0.0230), with the cessation of breastfeeding occurring around 6.3 years (marginal probability = 0.0331). The calculated difference between δ^15^N in breastmilk and δ^15^N in bone collagen averaged 0.9‰ (95% range), and the δ^15^N values of weaning foods averaged 9.2‰ (95% range). The WARN MDEs for Roman individuals suggest that weaning likely began at 1.2 years (marginal probability = 0.0331) and ended around 4.6 years (marginal probability = 0.0209). The difference between δ^15^N in breastmilk and δ^15^N in bone collagen was 2.8‰ (95% range), while the δ^15^N values of weaning foods averaged 10.6‰ (95% range).

### Adult population and diet reconstruction of Abdera population

**Fig. 5:**
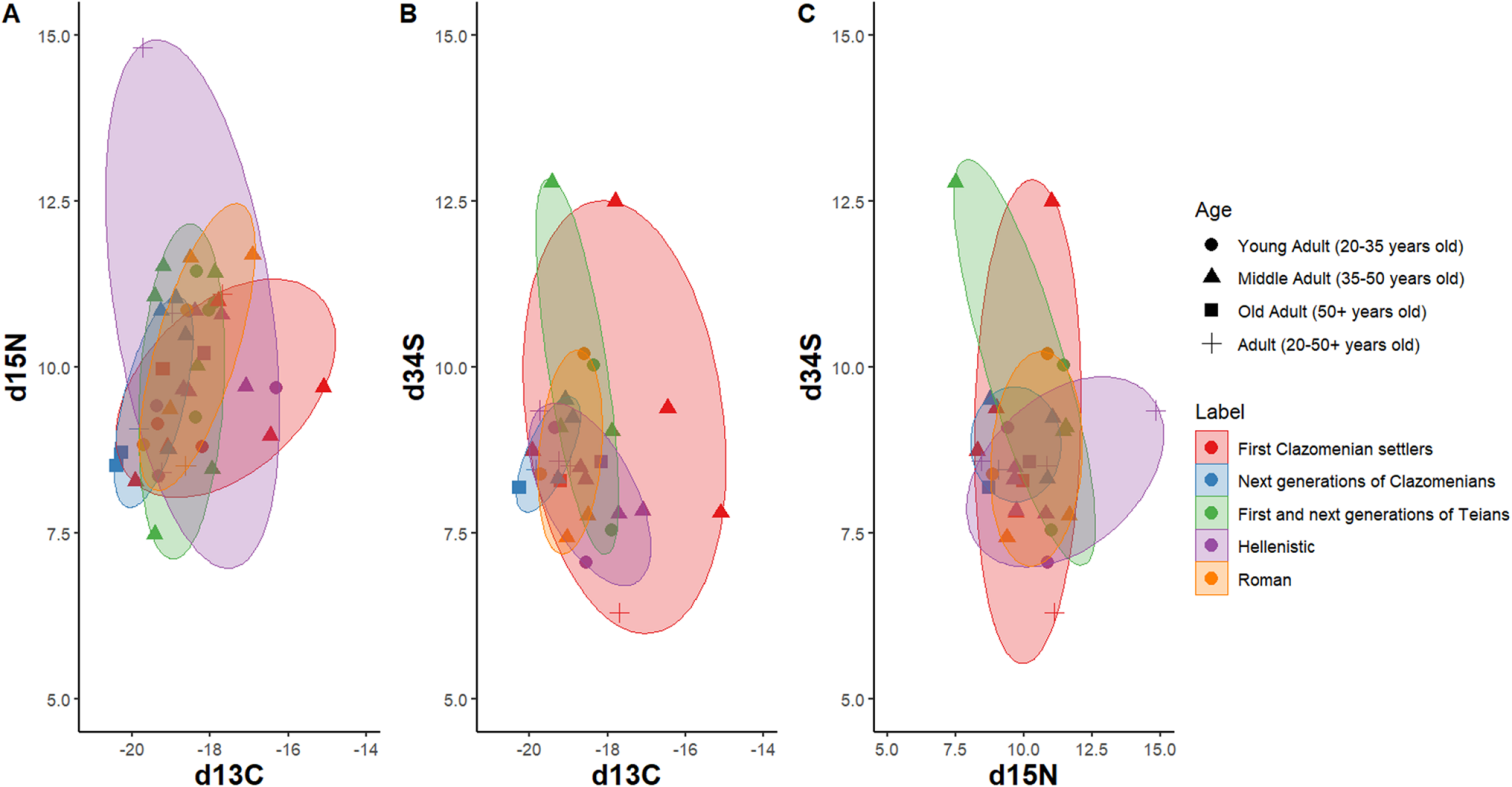
Scatterplots showing the spread of δ^13^C, δ^15^N and δ^34^S for Abdera adult individuals (n = 45) according to age groups, settlement phase and chronological period: first Clazomenian settlers: 654–570BC, Next generations of Clazomenians: late 6th c.–early 5th c. BC, Hellenistic cemetery: late 3rd c. BC–first half of 2nd c. BC, Roman cemetery: 200–400 AD)

Figure 5A illustrates the δ^15^Ν and δ^13^C of the adult population (n = 45), spreading from 14.82‰ to 7.48‰ and from −15.1‰ to −20.4‰, respectively. Among the 45 adult samples, 33 were also analysed for sulphur, with values ranging from 18.4‰ to −2.4‰. The age groups are distributed across the chronological periods rather than forming distinct clusters. The δ^13^C versus δ^15^Ν plot reveals the widest spread of data points among the groups. There is considerable overlap across all chronological periods, especially in Hellenistic and Roman periods while first Clazomenian settlers and Teians are more distinct. The δ^13^C versus δ^34^S (Fig. 5B) plot shows less variation and greater separation between groups. Specifically, first Clazomenian settlers exhibit minimal overlap, in contrast to the Hellenistic and Roman individuals. The δ^15^Ν versus δ^34^S plot (Fig. 5C) reveals the most compact clustering within each group and greater separation. However, due to the small sample size, statistical comparisons were not feasible.

**Fig. 6:**
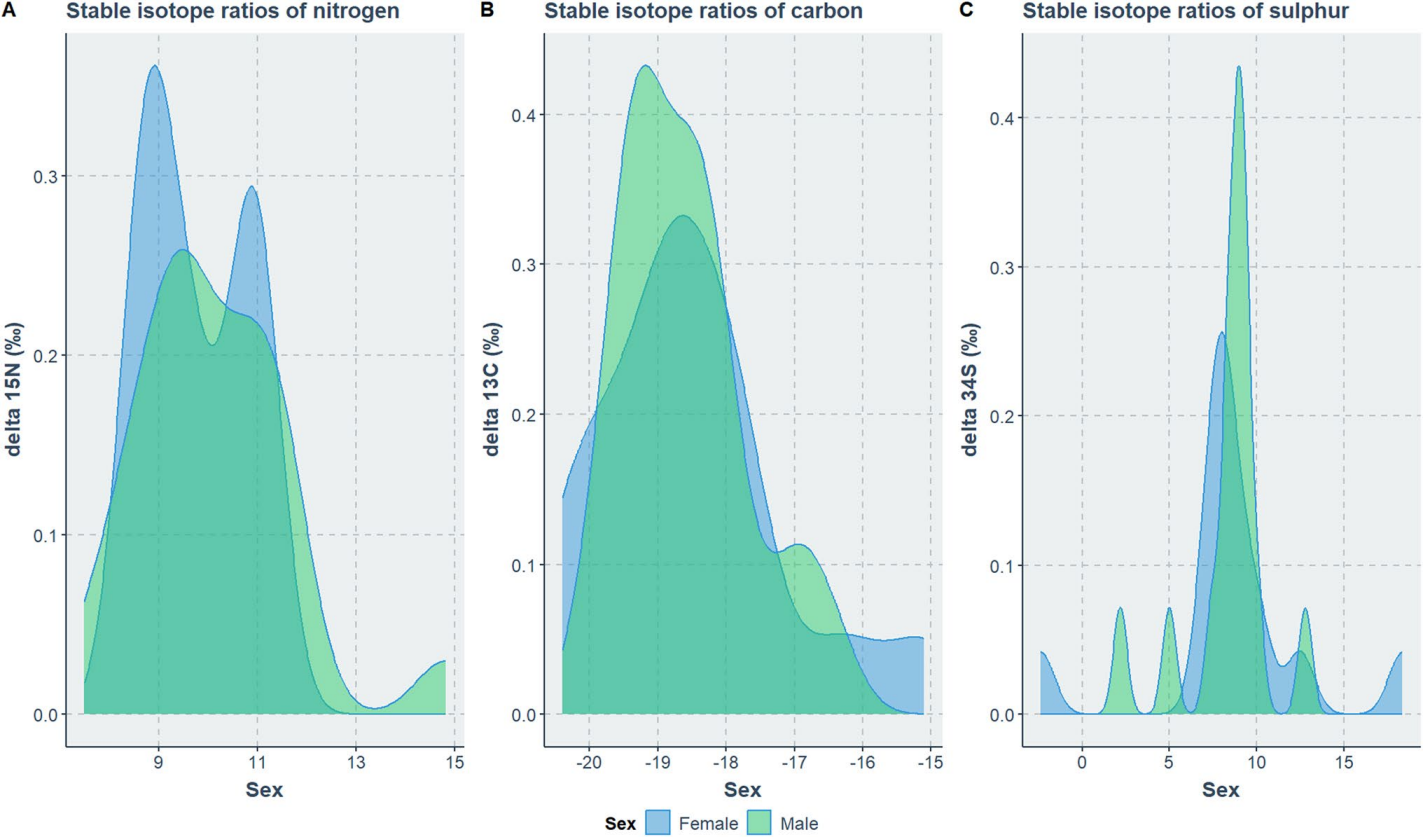
The distribution of δ^15^N (A), δ^13^C (B) and δ.^34^S (C) for adult female and male individuals from Abdera (654 BC–400 AD)

Figure 6 presents the distribution of carbon, nitrogen, and sulphur ratios among the adult population by sex. Due to the small sample size, statistical comparisons were not feasible. The distributions of males and females overlap indicating limited variation between the two sexes. Female values display bimodal distributions, indicating the presence of two or more peaks. This bimodality is particularly pronounced in nitrogen and sulfur ratios, suggesting greater variability within the group of females compared to males. The statistical comparison of the δ^15^N, δ^13^C and δ^34^S ratios between subadults and adults were not found statistically significant (δ^15^N: *p* = 0.35, δ^13^C: *p* = 0.75, δ^34^S: *p* = 0.62).

### Diet reconstruction using Bayesian modelling

#### Abdera population

**Fig. 7:**
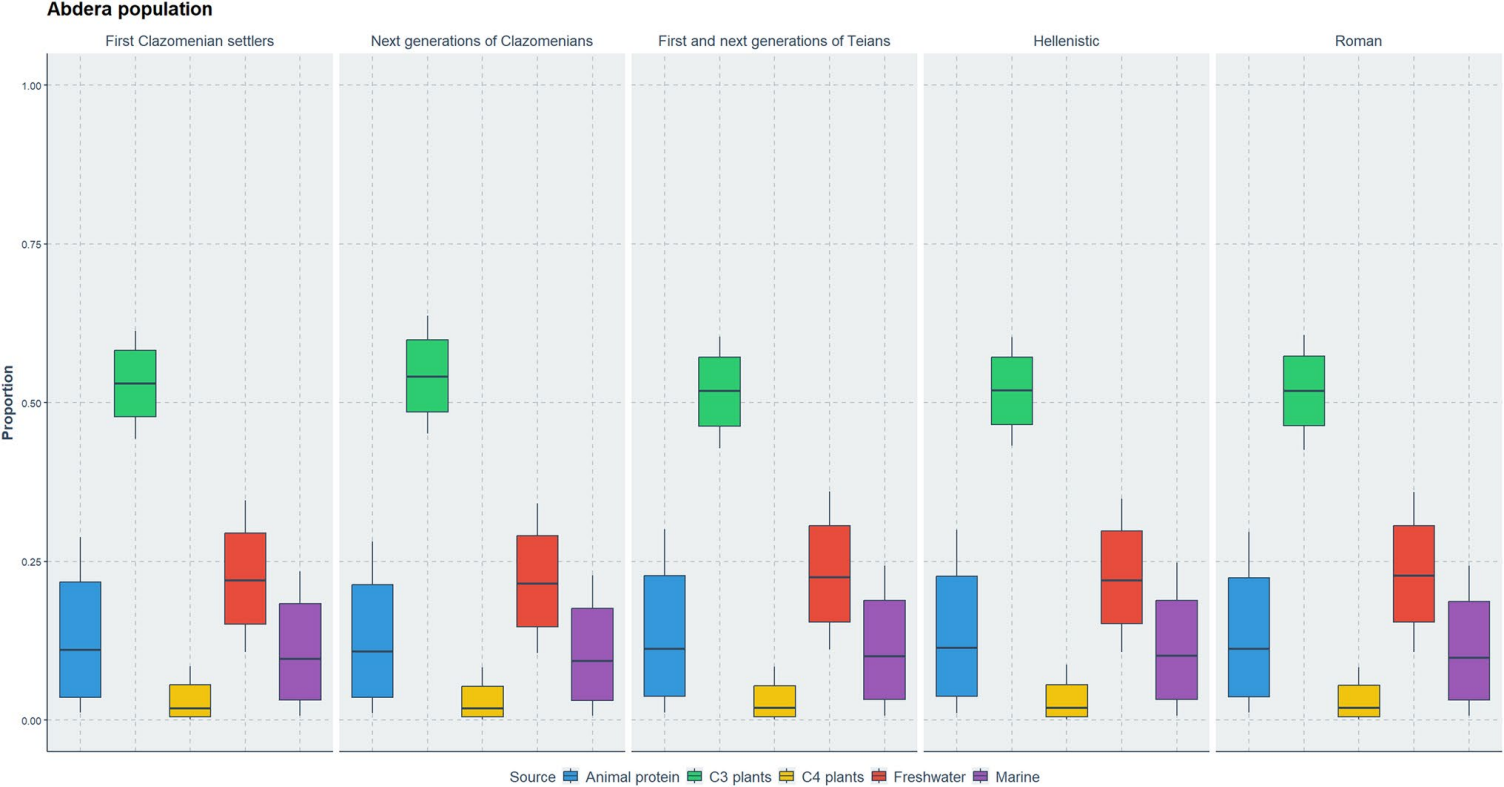
Boxplots showing the dietary proportions of foodstuffs (animal protein, C_3_ and C_4_ plants, fish) of the Abdera population obtained from MixSIAR according to settlement phase and chronological period: first Clazomenian settlers: 654–570BC, Next generations of Clazomenians: late 6th c.–early 5th c. BC, Hellenistic cemetery: late 3rd c. BC–first half of 2nd c. BC, Roman cemetery: 200–400 AD)

Figure 7 illustrates the dietary proportions of the Abdera population (Table S7) obtained from MixSIAR which show noticeable differences through the chronological periods. The proportion of animal protein remains consistently low across all periods, with some variability, but the narrow interquartile range (IQR) indicates stable, low levels. C_3_ plants dominate the diet in every period, showing the highest proportion across all groups, and the relatively narrow IQR suggests their consistent importance. In contrast, C_4_ plants maintain a consistently low proportion, with minimal variability, as indicated by the narrow IQR. Freshwater fish show moderate proportions with greater variability across periods, reflected in a wider IQR, suggesting fluctuating reliance on these foods over time. Marine fish, while also low, have slightly higher proportions than C_4_ plants and animal protein, with a narrow IQR indicating stable but limited reliance.

### Sulphur variation in Abdera

**Fig. 8:**
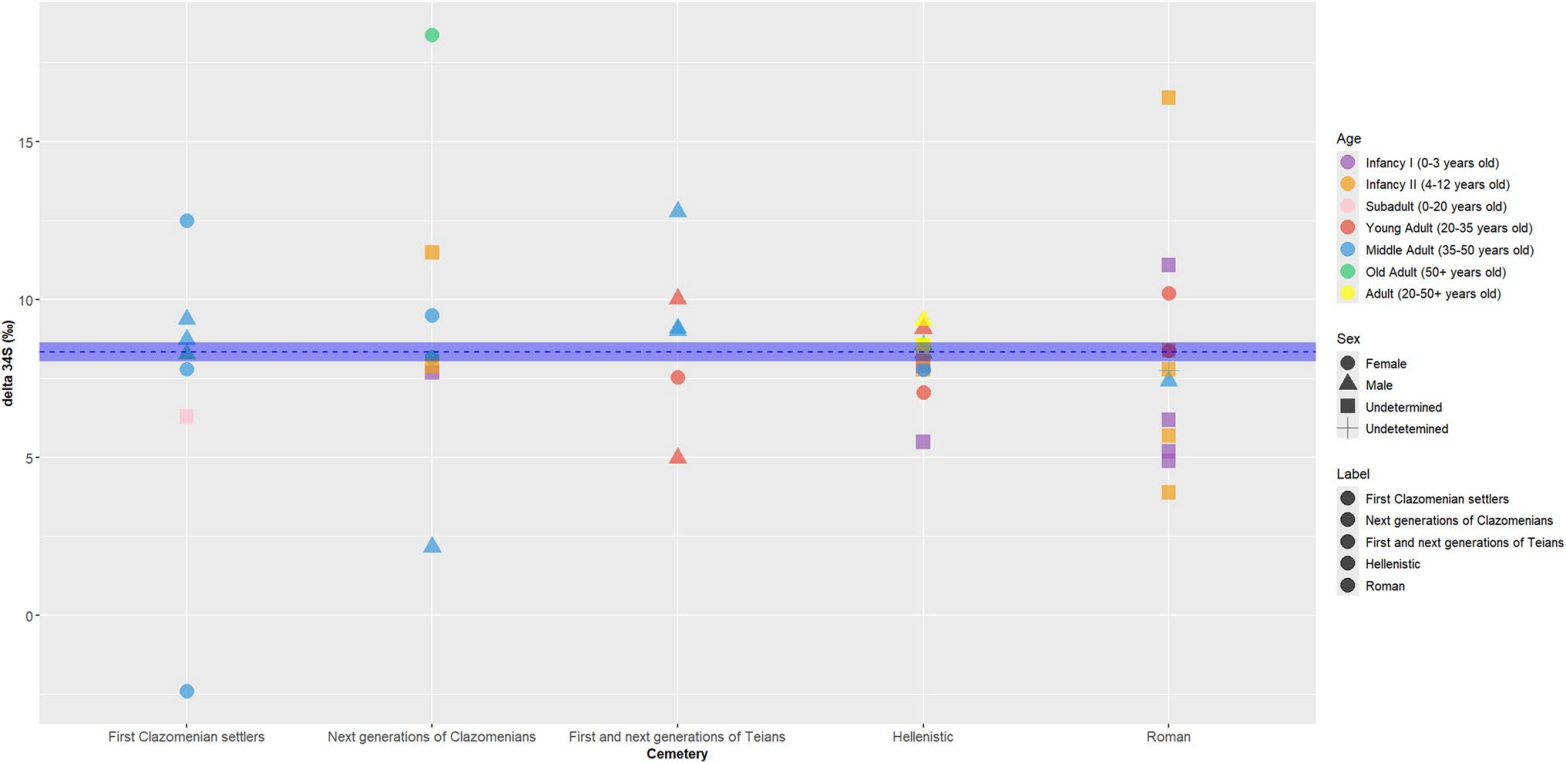
Plot showing the range of δ^34^S values for 45 individuals from ancient Abdera according to age groups, settlement phase and chronological period: first Clazomenian settlers: 654–570BC, Next generations of Clazomenians: late 6th c.–early 5th c. BC, Hellenistic cemetery: late 3rd c. BC–first half of 2nd c. BC, Roman cemetery: 200–400 AD)

Figure 8 illustrates the distribution of sulphur isotope ratios across groups, categorized by sex and age estimation, with the isotopic baseline of the site indicated by a blue dashed line. The Clazomenian individuals, including both the first and next generations, and the Roman individuals show the greatest variation, whereas the Hellenistic group exhibits more consistent values. Within the group of first Clazomenian settlers, two female outliers are observed. Similarly, the next generation of Clazomenians includes one female and one male outlier. The Teian group also shows variation, albeit to a lesser extent, with two male outliers (the predominant analysed sex in this group). The Hellenistic group is the most consistent, with only one subadult exhibiting more negative values. Lastly, the Roman individuals display significant variation, comparable to the next generation of Clazomenians, though most outliers in this group are subadults.

### Abdera and contemporary populations

**Fig. 9:**
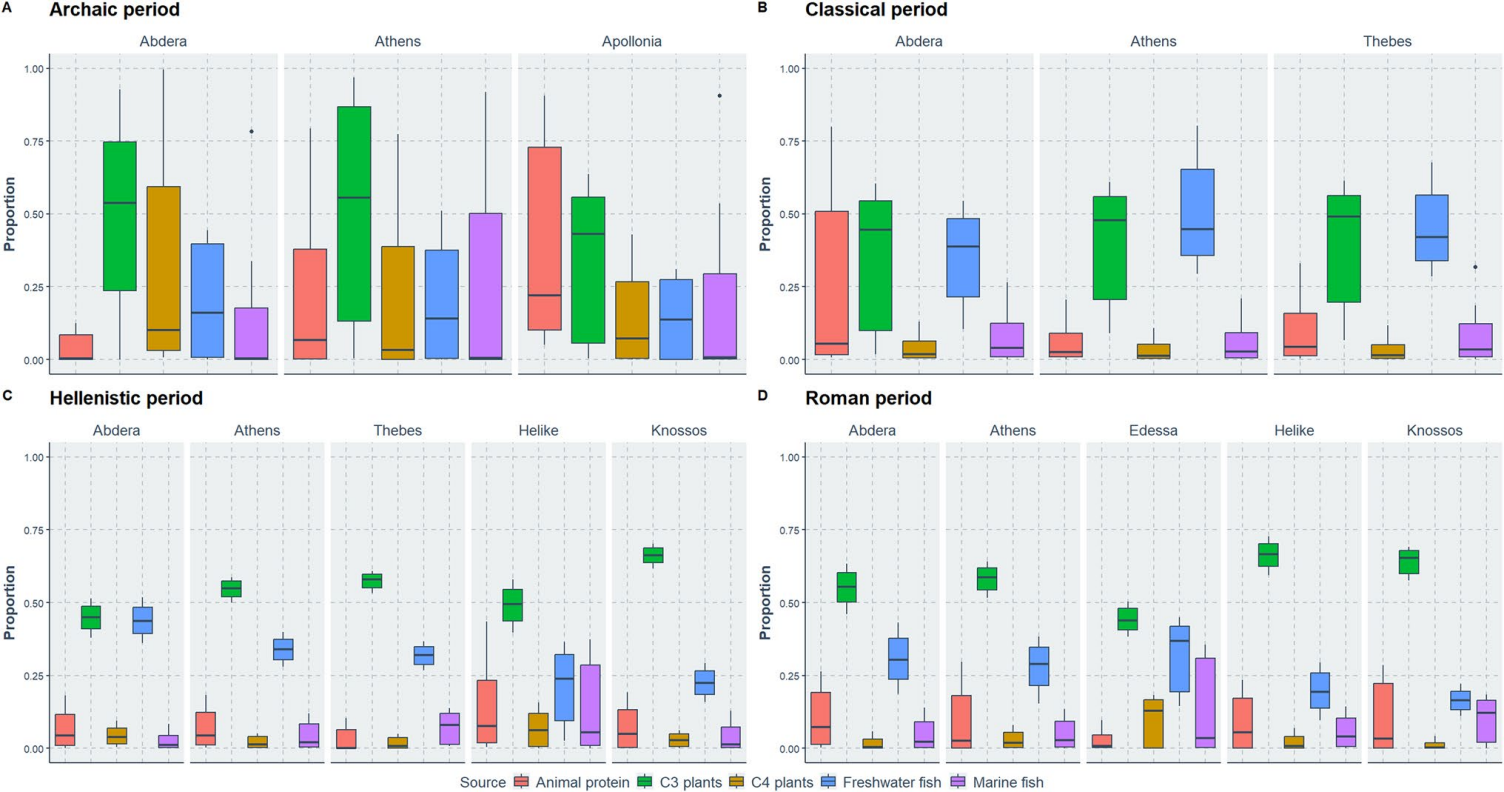
Boxplots showing the dietary proportions of foodstuffs (animal protein, C_3_ and C_4_ plants, freshwater and marine fish) of the Abdera population and other populations from ancient Greek cities presented per chronological periods (Archaic:700*–*490/80 BC, Classical: 490/80*–*323 BC, Hellenistic: 323*–*31 BC, Roman: 31 BC*–*4th c. AD). The proportions were obtained from MixSIAR

Figure 9 shows the relative proportions for different dietary sources in Greek colonies and ancient cities during the Archaic (Apollonia Pontica, Athens), Classical (Athens, Thebes), Hellenistic (Athens, Thebes, Helike, Knossos) and Roman periods (Athens, Edessa, Helike, Knossos) obtained from MixSIAR. Across all periods and sites, C_3_ plants and freshwater fish consistently dominate the dietary composition. Animal protein is moderately represented throughout, while marine fish and C_4_ plants remain consistently less significant. In the Archaic period, the dietary composition varies across Abdera, Athens, and Apollonia, particularly in the estimated proportions of freshwater and marine fish, while, C_4_ plants have greater estimates only in Abdera. During the Classical period, the dietary trends shift slightly in Abdera, Athens, and Thebes. In particular, freshwater fish in Abdera increase in equal components as C_3_ plants, while C_4_ plants and marine fish estimates decrease. These changes are observed in all other sites with the exception of animal protein, which shows a skewed distribution only in Abdera but reduced estimates in the other sites. In the Hellenistic period, the dietary patterns across Abdera, Athens, Thebes, Helike, and Knossos are consistent. C_3_ plants show high median values and variability. Freshwater fish and animal protein are moderately represented, while marine fish and C_4_ plants remain minimal. In the Roman period, the dietary composition across Abdera, Athens, Edessa, Helike, and Knossos exhibits similar trends. Freshwater fish emerge as the dominant component in all cities, with high median values and variability. C_3_ plants and animal protein are moderately represented, while marine fish and C_4_ plants remain minimal.

## Discussion

The encompassing question of this study was to investigate the dietary trends in subadult and adult diets in the ancient city of Abdera from its foundation as a colony of Ionians in the Aegean Thrace till the Roman period. For this purpose, carbon, nitrogen, and sulphur ratios from bone collagen was measured and two Bayesian models were employed to estimate probabilistic variations in dietary sources during adulthood (MixSIAR) and the duration of weaning (WARN).

### Different weaning strategies between Abdera's early establishment and Roman period

WARN estimates indicate that weaning in Clazomenian children began at 3.4 and was completed around 6.3 years old, representing a prolonged process compared to the colony of Apollonia Pontica but also to Roman period Abdera children, who were weaned at least two years earlier (Schmidt et al., 2016). This prolonged weaning estimation, combined with lower than expected, breastfeeding signal (~ 2–3‰), may indicate that the model’s calculations inherently estimate an extended weaning period due to the absence of isotopic values from infants younger than 4 years, which is the starting age of the Clazomenian subadults. Bearing this in mind, we infer that the prolonged weaning among the Clazomenians may reflect limited food availability during the early years of the colony’s establishment, forcing mothers to rely more heavily on breastfeeding as a nutritional source. Extended weaning periods have also been observed in Neolithic and Bronze Age populations (Fernández-Crespo et al. 2018; Stantis et al. 2020) and have been attributed to food shortages that restricted access to weaning foods. Previous anthropological research in the first colonial population of Abdera has reported elevated morbidity and mortality rates (Agelarakis 1999, 2010) especially in subadults, which aligns with our findings. Prolonged breastfeeding has been associated with increased child mortality (Sandberg et al. 2014) despite the recognized immunological advantages of breastmilk (World Health Organization 2009). Breastmilk alone does not fully cover an infant’s nutritional requirements beyond six months (Pérez-Escamilla et al. 2019) and delayed introduction to other foods may hinder immune system development (Fernández-Crespo et al. 2022).

By the Roman period, weaning began much earlier, around 1.2 years, and was completed by 4.6 years. The δ^15^N enrichment for this group was estimated at 2.8‰, aligning with the expected trophic level difference between breastfeeding infants and their mothers. Historical documents indicate that the Roman conquest led to severe resource depletion, ultimately driving Abdera into economic decline and obscurity (Kallintzi 2018). These circumstances may have strained the energetic intake of mothers, forcing them to introduce solid foods earlier rather than relying on extended breastfeeding. Moreover, Soranus, the most notable physician on obstetrics and paediatrics of Roman times, proposed shorter and earlier weaning ages (between 1.5–2.0 years) (Temkin 1991). According to many empirical studies his suggestions were widely applied across the Roman Empire (Ganiatsou et al. 2023; Velte et al. 2023; Fulminante 2015).

Both Clazomenian and Roman infants were weaned primarily consuming C_3_ resources. However, C_4_ plants (millet) was more common in Clazomenian infants and fish in 6/11 Roman individuals (ABD56, ABD52, ABD45, ABD46, ABD49, ABD55, Table S1). This pattern is similar to the diet of adults, which included millet during the early phases of the colony’s establishment, including weaning and fish during the Roman period.

Finally, it is important to note that while the WARN model remains one of the most effective methods for estimating weaning ages from bone collagen isotopic data, two limitations must be considered when interpreting the results. Firstly, the model provides population-level estimates, averaging data across individuals. Secondly, bone collagen may reflect physiological stress or growth rather than precise dietary shifts, such as the breastfeeding-weaning transition we aim to trace. We reckon that more sophisticated sampling approaches, such as the analysis of incremental dentine, offer more refined chronological resolution and therefore greater accuracy in assessing weaning (Beaumont, 2020; Beaumont et al., 2018; Fernández-Crespo et al. 2022). Nevertheless, our approach provided valuable insights into infant feeding strategies in Abdera, which can be refined in the future using incremental dentine sampling on a larger and diachronic dataset from the site.

### Diachronic consumption of plants and fish in Abdera over nine centuries

The isotopic ratios suggest that diet in Abdera remained relatively similar throughout a nine-century span, mostly based on C_3_ plants, such as cereals, grains and vegetables and fish sources (Fig. 7). The increased C_3_ group food consumption is strongly supported by historical sources and archaeological evidence. Farmsteads were established outside the city walls of Abdera as early as the 5th c. BC despite the hostile indigenous populations (Kallintzi 2011). The region’s fertile plains supported extensive vine and cereal cultivation. Historical records confirm the agricultural wealth of Abdera e.g. in 376 BC, the local tribe of Triballi, attacked Abdera due to grain shortages (Kallintzi 2012); Diod. XV 36, 1–4), in 170 BC the Roman general Hortensius demanded 50,000 modii of wheat (Kallintzi 2011; Liv. XLIII 4, 8–13), in second century BC a decree granted the Roman citizen Marcus Vallius the right to export 10 medimni of wheat annually (IThrAeg 202–204 (E 8). Finally, Abdera had a famous and extensive local wine production as evidenced by coins dating to 395–360 BC and local transport amphorae from the fifth and fourth centuries BC (Kallintzi et al. 2022).

Apart from C_3_ plants, C_4_ plants were also consumed in Abdera. The main representative of C_4_ plants in Greek antiquity is millet. Millet is known for its relatively straightforward cultivation and has been historically linked to lower social status and livestock feed (Garnsey 1999; Valamoti 2016). Usually, the increased carbon ratios are inferred as meat or dairy consumption from animals systematically fed with millet. However, in the case of Abdera the combination of increased carbon and low nitrogen ratios suggests that this signal is more likely derived from the direct consumption of C_4_ plants.

Apart from plants, fish was a common food component in Abdera. Based on the range of sulphur values, fish were most likely derived from freshwater environments. Fishing in lakes and rivers offers many advantages compared to the open sea and has been suggested by isotopic studies in the northern Aegean (Ganiatsou 2023). However, due to the lack of sulphur ratios from local waterbodies, it is not possible to identify the origin of the fish. Potential sources could have been the Nestos River and the lake Vistonida which were located close to Abdera and were abundant in fish according to the ancient texts (Aristotle, History of Animals VIII 13).

Marine consumption was also found in Abdera but in selective individuals, specifically females (ABD 119, ABD 83) and children from the Clazomenian (ABD 2, ABD 94) and Roman populations (ABD 56, ABD 52). Local marine exploitation in Abdera during the Roman times is confirmed by historical sources, such as Athenaeus, who mentions that cuttlefish and a type of red mullet called"kestreis"were fished between Abdera and Maroneia and archaeological evidence that indicated that oysters and other marine species became even more popular (Kallintzi 2011). However, due to small size restrictions it is not possible to conclude to the extent of marine protein consumption, but only to conclude it was sporadically consumed.

Meat and dairy consumption remained relatively low throughout the analysed periods in Abdera (Fig. 7 and Table S7). The proximity of the site to sea, lakes and river likely contributed to a widespread preference for fish instead of meat. In this way the people gained a nutritional dietary source, rich in iron, to compensate for the lack of meat in their diet. This is supported by the Bayesian modelling approach and aligns with historical sources, which suggest that meat was not systematically consumed in ancient societies (Heinrich et al. 2021).

### Dietary and migration patterns of Abdera females

Sulphur variation in the site is particularly observed in children and females, who also exhibit increased variability in both nitrogen and carbon ratios. Small sample sizes prevented statistical comparisons; however, the data indicate sex-based differences at the site of Abdera. In particular, most females exhibit the most extreme values, indicative of C_3_ and C_4_ plant and fish consumption, in contrast to males. These variations in isotopic ratios of Abdera females could be linked to the roles of women in food production, preparation, or consumption but also, to restricted access to certain food items such as meat. This pattern is also observed in Apollonia Pontica (Keenleyside 2008).

Apart from diet, sulphur isotopes have the potential to trace locality (Nehlich 2015; Vika 2009; Tritsaroli et al. 2022). In this regard, Abdera females exhibit greater migration patterns compared to males, especially in the Clazomenian and Roman groups. Ancient sources indicate that the Clazomenian attempt to colonize Abdera was comprised mostly of males (Kallintzi 2011), which strengthens our interpretation of identifying non-local females. This practice aligns with patrilocal exogamy, a system in which women marry outside their social group or locality, as they are not involved in land or status inheritance. Patrilocal exogamy dates back to the early Neolithic and Bronze Age Europe and has been verified through ancient DNA and strontium isotope analyses (Mittnik et al. 2019; Knipper et al. 2017; Sjögren et al. 2020; Furtwängler et al. 2020; Blöcher et al. 2023). Bearing this in mind, sulphur variation in subadults could stem either by variation in their mothers' migratory origins, transmitted through breastmilk consumption (Bzikowska-Jura et al. 2020), or from dietary patterns such as direct fish consumption, as supported by their carbon ratios (Halcrow et al. 2021). Although our study is limited by a small sample size of sulphur isotope baseline, we highlight the potential of strontium or ancient DNA analysis in these individuals for providing deeper insights into female mobility patterns.

### Variability in dietary preferences among Greek colonies, coastal and mainland poleis

Our dietary modelling of ancient Greek populations, particularly from southern Greece, reveals consistent patterns during the Archaic and Classical periods, with greater variability observed in the Hellenistic and Roman eras. C_3_ plants remained the dominant dietary component, supplemented mostly by freshwater fish, as seen across all studied cities. Although Thebes and Athens are mainland sites, and Abdera is located on the northern Aegean coast, they showed similar dietary trends during the Classical period (Fig. 9). While sample size limits generalization, this observation could motivate future research.

The observed dietary heterogeneity during the Hellenistic and Roman periods in our results corresponds with the significant social and political transformations of these eras, characterised by cosmopolitanism and extensive trade activities (Morris 2005; Scheidel 2003; Wiemer 2013; Price 1991; Oliver 2011; Archibald et al. 2011).

Our modelling outcome aligns with the original studies that note a shift toward plant-based diets in the Hellenistic period (Vika 2011; Lagia 2015), and in the cases of Helike and Knossos, increased consumption of animal and fish sources, respectively (Borstad et al. 2018). However, increased animal protein consumption in Knossos initially suggested by Moles et al. (2022) was not found. This discrepancy may arise from differences in interpreting diets using raw isotopic values compared to mixing models. While Moles' interpretation is scientifically valid, MixSIAR accounts for metabolic variations within populations, which raw averaged isotopic signals and skeletal evidence cannot address. Overall, our findings indicate an increased reliance on aquatic resources across all populations (Fig. 9), addressing previous challenges in tracing fish consumption through isotopic data in Greece (Panagiotopoulou and Papathanasiou 2015; Vika and Theodoropoulou 2012).

Another insight from the Bayesian modelling is the skewed distributions in dietary components, which reflect differential consumption within sites. For instance, the animal protein component in Classical-period Athens, reflects variability in consumption among the analysed individuals in conjunction with social status. The Athenian sample includes individuals from the cemetery of Kerameikos that included high-status residents, politicians, and war dead; the cemetery at Plateia Kotzia contained ordinary citizens and the cemetery at Laurion that served as the burial site for slaves from nearby mines (Lagia 2015). The same is observed in the case of animal protein and C_4_ plant consumption in Abdera across all time periods; thus, a certain level of sporadic consumption of these foodstuffs can be identidied diachronically. These examples highlight the potential of Bayesian modelling in tracing differential access and provisioning of foodstuffs compared to traditional interpretations of raw isotopic data.

It is also important to highlight the limitations of Bayesian models for ancient dietary reconstructions (Makarewicz and Sealy 2015), especially because their validity is contingent upon the quality and representativeness of reference data. In the case of zooarchaeological and paleobotanical isotopic baselines, the available data remain incomplete and unevenly distributed across different temporal and geographical contexts. This scarcity introduces potential biases, as it provides only a fragmented and, at times, skewed approximation of dietary composition (Schulting et al. 2023). As isotopic research must extend beyond visual data interpretation, continuous validation of these methodologies is needed for enhancing their precision and ensuring more reliable dietary reconstructions.

### Dietary similarities among Greek colonies

The modelled diets of Abdera and Apollonia Pontica reveal the same reliance on C_3_ plants and fish resources likely due to similar coastal and fertile mainland environments. This plant-based diet, which is rich in phytates and inhibits iron absorption, could contribute to the exacerbated prevalence of metabolic diseases, such as anaemia, found in both sites (Agelarakis 1999, 2000, 2001a, b, 2012; Zisis 2023; Keenleyside 2008; Keenleyside and Panayotova 2006) (Keenleyside and Panayotova 2006). However, systemic malnutrition would also increase nitrogen levels (Reitsema 2013; Fuller et al. 2005), which, in the case of Abdera, are present only in the Roman and Classical periods, and mostly absent among the first colonists (Clazomenians). This fact, together with the absence of significant differences in dietary patterns between the two colonial attempts, indicates that malnutrition alone was not the cause of the colony’s failure. On the contrary, our data supports a previous scenario that attributed the colonization failure to infant and childhood mortality due to harsh climate conditions and hostile interactions with the Thracians (Herodotus I: 168–169; Skarlatidou 2010; Triandaphyllos 2019).

## Conclusions

The study of Greek colonization is characterized as an intellectually challenging endeavor (De Angelis, 2009**)**, since it was a multi-layered phenomenon that lasted for a long time and covered vast geographic areas. Our study provides empirical data on diet in ancient Abdera using robust statistical analyses from its colonial phase through later years. We combined stable carbon, nitrogen and sulphur analysis, supported by two Bayesian models (MixSIAR, WARN) to estimate dietary proportion estimates and weaning durations.

We found that subsistence in the colony was based mainly on the exploitation of freshwater and marine fish, C_3_ plants and less animal protein, while there are consistent indications of sparse millet consumption across all examined chronological periods. Sulphur analysis indicates that female mobility was greater, particularly during the colony’s foundation. In Abdera, Clazomenian infants were weaned for prolonged periods, with weaning initiation being around 3.4 years. In contrast, Roman infants initiated weaning at least two years earlier (1.2 years). This suggests that weaning practices were flexible, shaped by both adaptive responses to harsh environmental conditions and sociocultural beliefs about infant feeding.

This study contributes to a deeper understanding of diet and subsistence in Abdera, one of the most iconic sites of the Greek colonization. Our upcoming research with a larger dataset will target a broader view of the political, cultural and economic aspects of the relations between the colonists and the indigenous populations (Malkin 2003), as well as in conjunction with the history of both the mother-cities and the native populations (Petropoulos, 2005).

## Supplementary Information

Below is the link to the electronic supplementary material.

Supplementary file1 (XLSX 205 KB)

## Data Availability

No datasets were generated or analysed during the current study.
